# Assessment of the Environmental Feasibility of Utilizing Hemp Fibers in Composite Production

**DOI:** 10.3390/polym17152103

**Published:** 2025-07-31

**Authors:** Denis da Silva Miranda, Douglas Alexandre Casetta, Leonardo Coelho Simon, Luiz Kulay

**Affiliations:** 1 Interinstitutional Graduate Program in Bioenergy, State University of Campinas, Campinas 13083-970, Brazil; d272501@dac.unicamp.br; 2 Department of Chemical Engineering, University of Waterloo, Waterloo, ON N2L 3G1, Canada; douglascasetta@tangho.green (D.A.C.); leonardo.simon@uwaterloo.ca (L.C.S.); 3 Department of Chemical Engineering, Polytechnic School, University of São Paulo, São Paulo 05508-220, Brazil

**Keywords:** hemp fibers, natural composites production, life cycle assessment, environmental management, prognostic analysis

## Abstract

This study investigated the impact of incorporating hemp fibers into composites for manufacturing industrial parts. The Global Warming Potential (GWP) of producing a traditional polymer matrix composite containing glass fibers was compared to that of producing a counterpart from natural hemp fibers. The investigation concluded that the partial replacement of synthetic fibers with biomass reduced the GWP of the product by up to 25% without compromising its mechanical properties. This study also quantified and discussed the GWP of intermediate products obtained from alternative routes, such as the manufacture of hemp stalks and pellets. In these cases, the findings showed that the amount of CO_2_ absorbed during plant growth exceeded the emissions related to soil preparation, farming, and processing of hemp stalks by up to 15 times, and the processing of row hemp bales into pellets could result in an even “greener” product. This study highlights the importance of using bio-based inputs in reducing greenhouse gas emissions in the materials manufacturing industry and concludes that even partial substitutions of synthetic inputs with natural fibers can show significant reductions in this type of environmental impact.

## 1. Introduction

Plastics are versatile materials that can be utilized in a wide range of applications—from simple, short-term products to those requiring high levels of sophistication, technical specifications, and long-term use. These attributes have significantly increased global plastic production, which rose from 2 million tons in 1950 to 460 million tons in 2019. This growth represents an average annual increase of 8.2% over the past seven decades [1,2].

The growing demand for plastics and other materials can present environmental risks if not properly managed. This is because most of these materials are still of synthetic (and generally fossil) origin, require significant amounts of energy during manufacturing, consume natural resources during their lifespan in ecosystems, contribute to global warming due to their complex production chain, and threaten ocean biodiversity [3]. Aware of these characteristics and specificities, business leaders, policymakers, and representatives of sectors involved in the international plastics production chain have advocated for initiatives that increase the efficiency of their transformation processes while also promoting the reuse, recycling, and reduction in the consumption of derived products. In this context, technologies that aim to replace fossil-based materials with their plant-based counterparts—partially or even completely, when technically feasible—deserve special attention. The aim is to produce plastic composites with high technical performance and a reduced environmental impact. As a result, fibers from various plant sources, such as jute, bamboo, kenaf, and hemp, have increasingly been utilized in the production of materials commonly found in the automotive, military, packaging, and construction industries [4,5]. Reflecting this trend, the share of plant fibers in Europe’s composite production revenues rose from 15% to 30% between 2013 and 2022 [4].

In this context, hemp fiber warrants special attention not only for its excellent mechanical properties [6,7,8] but also due to the minimal requirements for its cultivation [9,10]. The agricultural processing of hemp is regarded as multipurpose, as it can produce seeds, fibers, or both on a large scale. Consequently, around 25,000 consumer goods are made by nine manufacturing sectors—agriculture, textiles, recycling, automotive, furniture, food and beverages, paper, construction materials, and personal care [11,12,13]. In the transportation industry, hemp fibers can be transformed into molded parts for passenger and cargo vehicles, with the benefit of being lighter than those traditionally made from glass fiber and polypropylene (PP) composites [7,10]. This is because the use of hemp fiber in the formulation of composites can reduce the mass of the materials by half due to their low specific weight [6,14] without compromising essential functionalities such as stiffness, strength, and durability [15,16].

Understanding the potential to reduce the environmental impact of using hemp in industrial processes is the focus of several studies reported in the scientific literature. A common feature of many analyses is the adoption of a systemic perspective, described through the life cycle concept, or the direct application of the Life Cycle Assessment (LCA) technique to quantify the effects on the surroundings [17,18,19]. Also, in most instances, the environmental impact profile is limited only to the Global Warming Potential (GWP) of the arrangements investigated. After investigating the Global Warming Potential (GWP) of substituting glass fibers with hemp-based fibers in composites for vehicle part manufacturing, Zarafsh ani et al. (2023) [20] concluded that applying plant biomass could decrease GHG emissions from motorcycle production by up to 14% and those associated with the life cycle of aircraft by approximately 68% [21,22,23]. Heidari et al. (2019) [24] found that incorporating hemp into concrete for constructing housing walls can lower the GWP related to the product by 81% compared to that achieved through conventional construction techniques [21,22,23]. Other studies have also indicated significant reductions in GHG emissions from the life cycles of various products when glassy and polymeric materials are replaced by hemp fiber-based intermediates without compromising mechanical properties [21,22,23].

Hybrid composites made from engineered polysaccharides and glass fiber can produce lightweight polypropylene materials that offer environmental benefits while meeting automotive industry standards. Specifically, polysaccharides (such as starch, glucan, and cellulose) reduce weight, which directly lowers the energy needed for vehicle operation. Ganesarajan et al. (2022) [25] showed that a polypropylene hybrid composite with an engineered polysaccharide and glass fiber had an environmental footprint of 3.06 kg CO_2 eq_ significantly less than the 3.51 kg CO_2 eq_ of similar polypropylene composites with only glass fiber [21,22,23]. Following this approach, this study aims to assess the environmental impact of a composite made from hemp fibers grown in Ontario, Canada, combined with a polypropylene matrix, for potential use in plastic structural components.

This project is part of a process development program, whose initial phases—technological development and economic feasibility studies—have already been completed. In this case, hemp fibers are produced through direct microdecortication and pelletization [26] instead of traditional bast fiber decortication methods. This new method aims to create short hemp fibers more efficiently, though its environmental impact has not yet been evaluated. Therefore, the Global Warming Potential (GWP) of manufacturing a traditional fiberglass and polypropylene composite is compared to that of producing a similar material using short hemp fibers processed with this new microdecortication method. Impact assessments are conducted using an LCA with a cradle-to-gate approach. This study also examines and discusses the GWP of intermediate products, such as hemp bales and hemp pellets. The findings are expected to support the recognition of hemp fibers as an environmentally sustainable alternative, judiciously replacing glass fibers in products designed with the principles of the Design for Environment (DfE) concept.

## 2. Materials and Methods

### 2.1. Specifications of the Study Object

**Figure 1:**
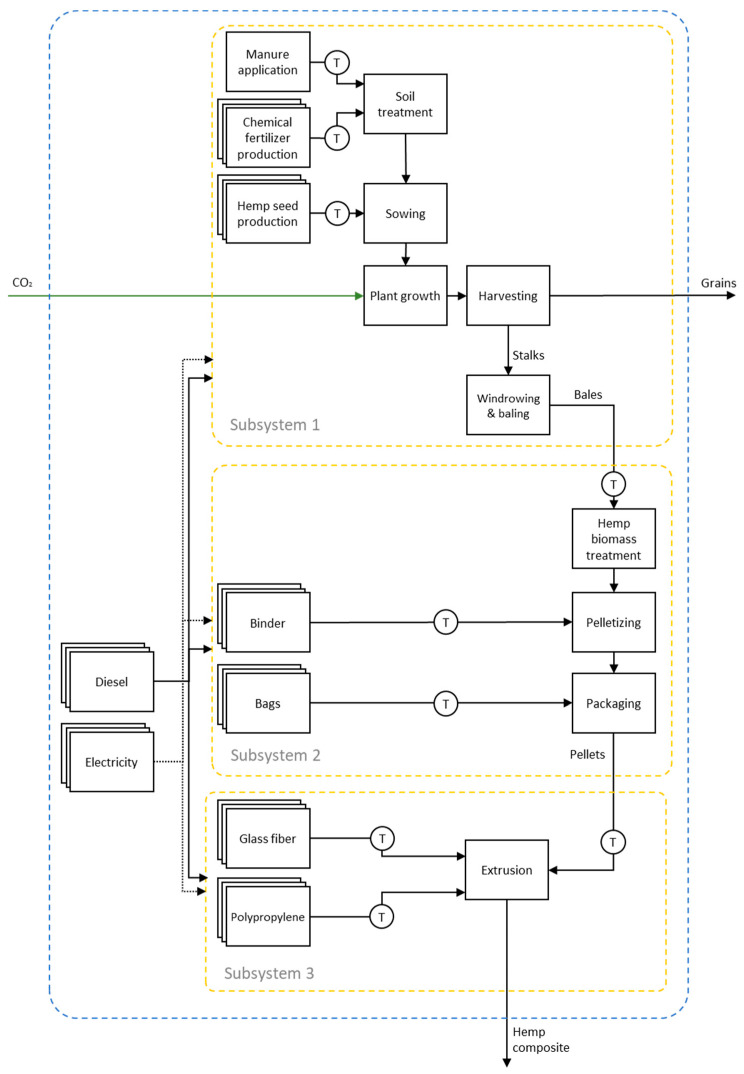
Global Product System for producing composites that include hemp fibers in the formulation.

An LCA was applied to three products derived from hemp processing: (i) hemp bales, (ii) hemp pellets, and (iii) hemp composites. The following sections provide detailed descriptions of the production processes of these products. This study included the transformation operations for each life cycle, which are interconnected through energy and/or material flows (Figure 1). To accurately characterize each production setup and avoid double-counting, the overall Product System was divided into three smaller production subsystems: Subsystem 1, which involved hemp stalks in bales; Subsystem 2, which involved hemp pellets in bags; and Subsystem 3, which involved hemp composites in pellet form. In Figure 1, the dashed lines represent the (sub) systems’ borders, the dotted arrows represent energy flows, and the solid arrows indicate material flows. The overlapping rectangles illustrate the life cycles of the inputs involved in each transformation, with their origins linked to the extraction of natural resources (cradle), which are consumed in these processes. Although these sequences are shown in a condensed form in Figure 1, all their operations were considered to estimate the environmental impact.

#### 2.1.1. Subsystem 1: Hemp Stalks in Bales

**Figure 2:**
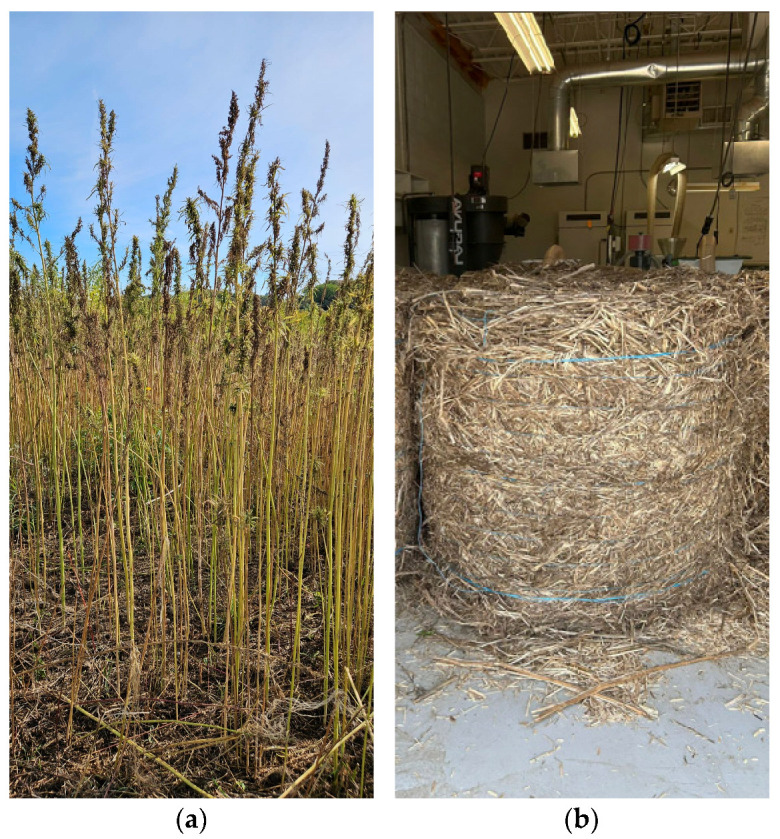
(**a**) Industrial hemp plants. (**b**) Bale of industrial hemp.

Subsystem 1 involved hemp cultivation, which produced hemp grains and stalks in bales, as illustrated in Figure 2. The crop chosen for this study was planted on a 66-acre plot in 2023 and a 32-acre plot in 2024, both located on the main campus of the University of Waterloo (ON), Canada. This situation meant that some parameters could not be determined from primary data. This gap was filled by values gathered from the literature, which were similar to and consistent with those of the operations practiced. The agricultural process began with soil preparation for planting, incorporating cattle and/or horse manure.

This study excluded the environmental impacts of acquiring these primary macronutrients and organic matter sources, as this practice corresponds to repurposing livestock waste. Thus, only the consumption and emissions related to transporting materials for 29 km from the supplier to the field were considered. The application of manure and subsequent plowing were conducted in separate stages using agricultural machinery that operated on diesel fuel.

Ontario’s well-drained, slightly alkaline clay soils (7.0 < pH < 7.5) are ideal for growing hemp. However, additional N-P-K supplementation beyond what manure provides is necessary to enhance agricultural productivity. This was accomplished by dosing a compound chemical, monoammonium phosphate (MAP), with a (52–11–0) ratio. The environmental impacts of fertilizer processing were also modeled using data from the Ecoinvent^®^ Database 9.5, which was adapted to reflect the specific conditions under which they occur. As with the other inputs in (successive) hemp fiber processing, MAP was examined using a cradle-to-use approach.

Therefore, in addition to the application of this fertilizer, which involved plowing with agricultural machinery, the industrial production of this fertilizer was also taken into consideration in the analysis. A plowing stage using discs (discing) was carried out before sowing the seeds into the soil. Organic hemp seeds were sourced from Winnipeg (MB, Canada), located 1996 km from the cultivation site. Like the other inputs, they were transported by road.

The lack of consistent primary data on hemp seed production led to this stage of the Product System being modeled after organic corn production. According to records from Harvest New York (2017) [11], the replacement by the substitute is consistent and representative of the conditions under which processing occurs in Winnipeg (Canada). Sowing was carried out mechanically, with 33.6 kg of seeds per hectare being applied at this stage of cultivation.

The amount of CO_2_ absorbed and stored in the hemp biomass during plant growth was determined using secondary data. For this purpose, seven studies published in the literature were utilized; the selection criteria were based on the similarity of the technological and operational aspects of the crops reported in the references to those practiced in Ontario. The CO_2_ uptake rates achieved in each situation are presented in Table 1, along with their respective sources. This series estimated an average value of 1.69 kg of CO_2_ fixed per kg of biomass produced. The stages following plant growth—grain and stem harvesting and baling—were performed with the assistance of agricultural machinery.

Since the harvesting process yielded two products, it established a multifunctional situation. In conceptual terms, the allocation procedure should address cases of multifunctionality in LCA applications within the attributional modality [34], as in the present case. The application of this approach will be discussed later in Section 2.2.

During harvesting, the biomass may be left in the field in windrows for a few days or weeks to facilitate the retting process. Retting may be used as a method to reduce the energy required for the traditional decortication of long hemp fibers, which is typically achieved using conventional methods with hammer mills. However, the technique used here was microdecortication. This will be further discussed in the next section. The GHG emissions of this operation were negligible when verified using a cumulative mass contribution criterion, MCC = 1.0%, which was defined for data treatment during Product System modeling [34]. The agricultural stage concluded with the formation of hemp bales.

#### 2.1.2. Subsystem 2: Hemp Pellet Production

**Figure 3:**
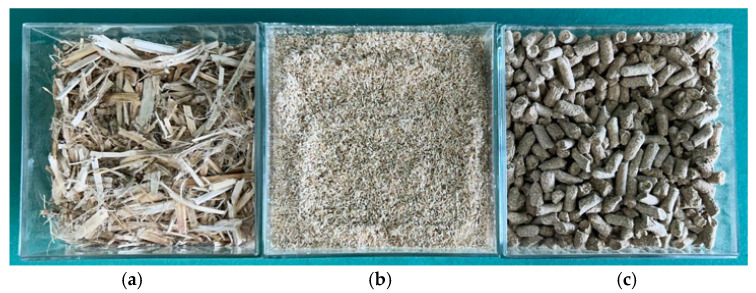
(**a**) Hemp fibers. (**b**) Milled hemp fibers. (**c**) Hemp pellets.

The next phase of the production arrangement involves transforming hemp bases into pellets (Figure 3). Subsystem 2 essentially consists of three operations: milling, pelletization, and packaging. The milling operation involves particle size reduction, taking a bale of hemp stalks as input to produce short hemp fibers.

The method of particle size reduction (milling) used here is based on newly invented technology known as microdecortication [26]. It produces short hemp fibers (less than approximately 2.0 cm or smaller) utilizing the entire hemp stalk from the bale. Microdecortication utilizes cutting mills, which produce a high yield of short hemp fibers (>85%), and the use of retting may not be necessary. This is fundamentally different from the conventional method of decortication (using a hammer mill), which separates long hemp bast fibers (usually more than 20 cm long, approximately 20% (w/w)), hemp hurds (about 70% (w/w)), and dust. The newly invented method of microdecortication is far more efficient because the production yields of short fibers are in the range of 85% or above. It has already been demonstrated in a pilot plant (operating at less than 100 kg per hour) at the Tangho Green Canada Technology Center in Waterloo, ON, Canada; however, it has not yet been implemented in practical, commercial terms (hundreds of kilograms per hour). Therefore, this compelled us to use secondary data obtained from mass and energy balances conducted under assumed regular commercial operating conditions.

The second operation of Subsystem 2 is pelletization. This operation utilizes equipment that compresses biomass inside a die to produce a compacted pellet. An advantage of handling short hemp fiber pellets instead of losing short fibers is a reduction in bulk density.

This strategy lends a prospective character to the environmental diagnosis made in this study for Subsystem 2. The results obtained by applying an LCA can inform Design for Environment (DfE) actions, aiming to develop the hemp pellet production process to minimize impacts related to the Global Warming Potential.

**Figure 4:**
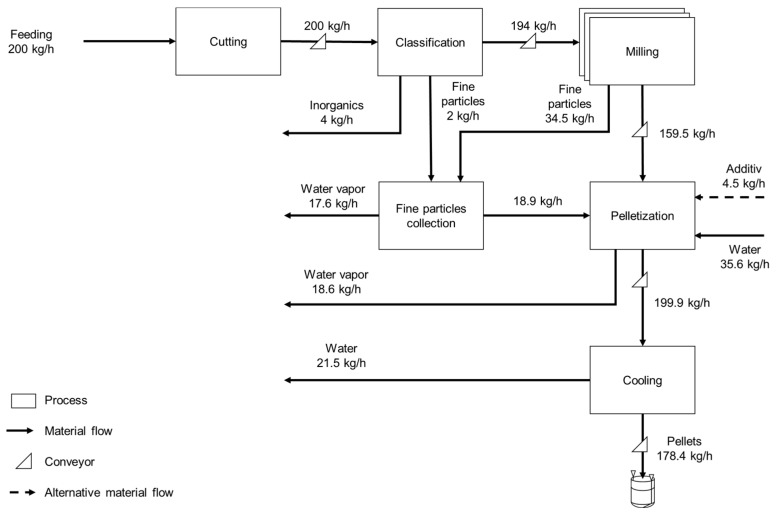
Main mass balance streams of the hemp pellet production process adopted for modeling the Product System.

All flows entering and exiting the Product System boundaries were designed to handle 200 kg/h of hemp bales. The unavailability of specific consumption and emission values necessitated their determination through mass and energy balances, considering the conditions and performance rates outlined in the literature. Figure 4 illustrates a process flow diagram, emphasizing the quantities of the main flows.

In this stage, the bales produced in Subsystem 1 are transported by road to Processing Site 1, located 1000 km from the plantation. The first operation is named cutting, which aims to disassemble the large bale into chips (>5.0 cm). The cutter’s reception, storage, and feeding operations are performed manually. The cutter converts the hemp bale into short stalks and chips, which are transported using a conveyor belt. Sorting and sieving separate waste mixed with biomass (stones and sand), which accounts for approximately 2.0% of the total mass of the bales. The fines generated from hemp stalks during sorting are collected for later reuse.

The sorted material is comminuted in successive stages of particle size reduction and fractionation (presented as cutting, classification, and milling in Figure 4), leading to the generation of short fibers with a desirable particle size distribution. The short hemp fibers are subsequently transferred for pelletization. Comminution also produces fine particles, which are combined with those generated during sorting, after being captured by a system that includes a collector and a bag filter, to be reintroduced into the pelletizing process. Pelletizing is conducted wet, and a binding agent may be added to enhance the process efficiency, depending on the specific conditions. For clarification, the pellets produced in Subsystem 2 contain short hemp fiber, fines, moisture, and a small quantity of additives. Note that the term “pellet” is used for the products of Subsystem 2 and Subsystem 3, but they differ from each other. Finally, the hemp pellets are cooled and bagged in polypropylene bags with a storage capacity of 2.0 m^3^ and an estimated unit mass of 2.5 kg.

The energy balance of Subsystem 2 was conducted based on the nominal capacities of the equipment utilized in the process arrangement, using data and information provided by Tangho Green Canada Inc. The differences between the dimensions of the values that guided the preparation of Subsystem 1 and those reported by the company were addressed by linearizing the latter set to align with the processing flow defined for the analysis (200 kg/h). The electrical energy consumed by each piece of equipment is shown in Table 2.

#### 2.1.3. Subsystem 3: Production of Composite Containing Hemp Biomass

**Figure 5:**
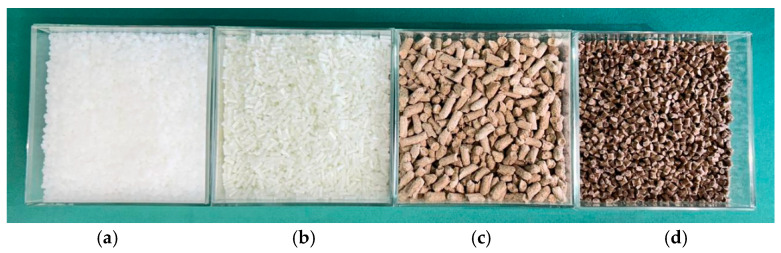
(**a**) Polypropylene. (**b**) Polypropylene with glass fiber. (**c**) Hemp pellets. (**d**) Composites: 70% polypropylene and equal parts (15:15)% (w/w) of glass fiber and hemp pellets.

Subsystem 3 comprises the manufacture of a hybrid composite (Figure 5). This material is produced by extruding a mixture that includes the pellets processed in Subsystem 2, glass fiber, and polypropylene resin. The composite has been made on a pilot scale (several kilograms per hour). Still, it has not yet been manufactured on a large commercial scale, thereby allowing for a prospective assessment of Subsystem 3’s environmental performance. These results can also inform future Design for Environment (DfE) actions aimed at reducing its Global Warming Potential.

The design of the hybrid partially natural composite was founded on a fundamental premise: its technical performance should be compatible with that achieved by the existing substitute in the applications where those materials are utilized. The automotive sector, where polypropylene composites are often employed to manufacture interior and exterior parts for the reinforcement and protection of molded structures, has proven to be a compelling scenario for validating this requirement because their mechanical properties and specifications are rigorously evaluated before they become vehicle components.

Consequently, parameters such as the tensile and flexural moduli, impact notch, and density were established as criteria for selecting the natural composite. Following extensive research, we developed a hybrid composite comprising 70% (w/w) polypropylene and equal parts (15:15)% (w/w) of glass fiber and hemp pellets. If successful in both technical and environmental domains, this variant could replace a similar material used by automakers to produce car parts, whose formulation consists solely of polypropylene and glass fiber in a [70:30] mass ratio.

Table 3 illustrates the average performance obtained by both materials during the analysis. These results confirm the initial expectation that the mechanical properties of the alternative composite would be maintained despite 50% (w/w) of glass fiber being substituted with short hemp biomass. Consequently, the alternative composite became the product of Subsystem 3, and this step in the study focused on it. Furthermore, its environmental validity regarding the GWP could be compared to the performance of the existing composite, which was used as a reference for analysis.

**Figure 6:**
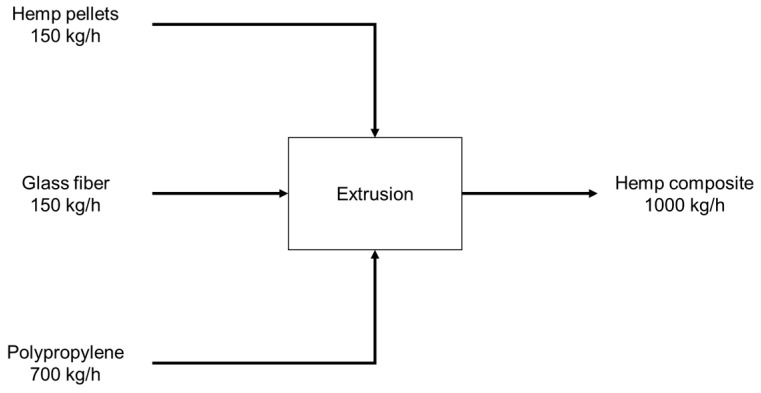
Mass balance for the hybrid composite production process with glass and short hemp fibers.

This stage of the Product System began by transporting the three active ingredients in the formulation to the unit where the composite is processed. The plant is located 500 km from the hemp pellet and glass fiber supply depots and approximately 100 km from the PP resin distributor. Composite processing was modeled exclusively on the extrusion and pelletization of hybrid composites (final gate) but did not include injection molding. Figure 6 displays the values of the material streams used to estimate the Global Warming Potential of the process. A TSE-95 extruder from Nanjing Haisi Extrusion Equipment Co., Ltd. (Nanjing, China) was selected as a representative of the compounding stage. It features a twin screw, a variable load capacity of 800 to 1200 kg/h, and an electrical power of 315 kW, which could be used to estimate its environmental performance.

The selected TSE-95 model is also compatible with the anticipated installation size for producing the natural composite. The extruder feeding and product packaging were lumped with extruder inputs, which means that the environmental loads associated with these processes were no longer included in the environmental performance analysis.

### 2.2. Life Cycle Modeling

#### 2.2.1. LCA Scope Definition

The determination of the Global Warming Potential of the production of hybrid composites containing short hemp fibers, along with their intermediates—hemp in bales and hemp pellets—through the LCA was based on the methodological standardization outlined in the ISO 14040 and 14044 standards [34,35]. The technique was applied in an attributional modality and followed a “cradle-to-gate” scope. The intensities with which the transformation steps of the composite processing were executed were directly linked to the properties that this production good must demonstrate to fulfill the technical requirements for serving as a structural material in automotive panel manufacturing. Given its linear arrangement, the same conditions also guided the production of the intermediates. Similarly, a comparable approach was used to produce the reference composite made of PP and glass fiber.

The reference flows (RFs) adopted in each case to conduct the LCAs included 1.0 kg of hemp stalks in bales (Subsystem 1), 1.0 kg of hemp pellets in bags (Subsystem 2), and 1.0 kg of hybrid composite (Subsystem 3 and reference arrangement). As mentioned in Section 2.1, this study used secondary data. These data were either collected from the technical literature and datasets in the Ecoinvent^®^ Database 9.5 or provided by companies in the hemp fiber production and manufacturing sector. Following the guidelines of ISO 14044 [35], the modeling of the subsystems was carried out, considering the quantitative criteria of the cumulative mass contribution (CMC) and cumulative energy contribution (CEC) to exclude environmental aspects. For the arrangements under analysis, CMC and CCE values of 1.0% were applied. Additionally, the consumption of materials and energy, as well as emissions, below these limits was evaluated for its potential environmental relevance [35] and reintegrated into the analyses when necessary.

The environmental impacts associated with polypropylene resin and glass fiber production were quantified using secondary data from the Ecoinvent^®^ Database 9.5, which were adjusted for the research conditions. The same strategy was applied to the assets comprising the operational recipe of the analyzed composites, as well as the process utilities (e.g., electrical and thermal energy and water) and transport operations.

Regarding geographic coverage, the study encompassed the Canadian provinces of Manitoba, where hemp seeds are produced; Ontario, the region where the plant is cultivated; and a location 1000 km away from Waterloo, where the pellets and composites are made. We also assumed that the reference composite would be manufactured in the same areas to avoid disparities and distortions in the results. This study’s temporal coverage primarily consisted of 2023. The exception was the diesel oil consumption of the agricultural machinery and means of transport, which was recorded for 2019, as this period typically represented such demands. Finally, the technological coverage was specified based on the technological and technical aspects of each subsystem, as described in Section 2.1. Lastly, concerning utilities, data and information about the electricity sources required for the production processes were gathered from the Ecoinvent^®^ Database 9.5 and updated for temporal coverage. For the fuel used in the process, estimates of the consumption, origin, destination, and import volume were cross-referenced with data from the Canada Energy Regulator 2024 [36] and customized for the locations included in this study.

Whenever a given operation (a process or a set of processes) receives various inputs, generates co-products (outputs) that are intended for potentially different functions, or is even connected to a more extensive arrangement such as open-loop recycling (OLR) that serves more than one use simultaneously, the LCA practitioner will be faced with a multifunctional situation. Cases like this should be treated to distribute the environmental loads associated with the multifunctional transformation, and cases with processes involving co-products should be treated to distribute those generated in the operations before it [37]. The allocation procedure is adopted with artifice for attributional LCA studies to treat multifunctionalities. In general terms, this consists of applying a partition factor estimated from physical relationships or, when this is not possible, applying one estimated from the economic value of the inputs (or co-products) that coexist with the environmental loads related to the process under treatment [38].

In this study, this situation arose in the agricultural stage (Subsystem 1), specifically during harvesting, when hemp stalks and grains were produced. We decided to investigate the effects of the multifunctional situation between these co-products by applying the physical mass criterion. Consequently, the environmental loads generated were divided proportionally to the quantities of hemp stalks and grains obtained in this process stage.

Given the objectives of this study, we adopted the method proposed by the Intergovernmental Panel on Climate Change (IPCC) version 1.02 for 100 years, including CO_2_ uptake [39], to quantify the Global Warming Potential associated with each analyzed situation. As the title indicates, the method considers the CO_2_ absorption rates of the photosynthetic processes of various anthropogenic crops throughout their growth cycles.

Additionally, it addresses biogenic emissions related to the natural carbon cycle and those arising from anthropogenic activities, such as combustion, harvesting, digestion, and fermentation, which lead to the decomposition or processing of bio-based materials. Biogenic CO_2_ is produced when the carbon in biological matter reaches its highest degree of oxidation due to these interventions [40,41]. The CO_2_ absorption and emission rates during a short cycle, without deviations, will be complementary, resulting in a net zero impact on the GWP. However, this balance can be disturbed if biogenic carbon is sequestered by a product intended for use in another life cycle or when it is emitted in its reduced form (e.g., methane). Regardless of the established condition—equilibrium or instability—the IPCC 2021 GWP100 (including CO_2_ uptake) can estimate the effects of these deviations in terms of the GWP [39].

#### 2.2.2. Complementary Assumptions

In addition to the requirements for defining the scope outlined in Section 2.2.1, further assumptions were established to model the subsystems under analysis. These are detailed below:

- The analysis excluded environmental loads from the production of capital goods and labor usage because their contribution rates were minimal, and the data associated with them were significantly uncertain.
- All transportation operations were modeled considering that EURO 6 trucks would be used, featuring load capacities between 7.5 and 16 tons.
- Also, regarding transport, only the atmospheric emissions from fuel combustion during truck movement were considered. Consequently, this study excluded other environmental loads from constructing and maintaining roads and vehicles.
- Data on electricity generation sources and their respective shares in each location within the geographic coverage were also obtained from Ecoinvent Database 9.5 and updated for 2023.
- Mass and energy balances were applied to the data to verify their consistency and representativeness and to harmonize them, reducing discrepancies arising from the diversity of sources.

After several adjustments, the subsystem models were found to be suitable for a prospective environmental performance assessment, as planned for a product still in the design and development phase.

#### 2.2.3. Sensitivity Analysis

After obtaining the environmental impact results, a sensitivity analysis was conducted for key variables of the Product System. These parameters were selected because they were under the control and management of the composite formulators. The criterion was established to reduce the deterministic nature of the modeling and to allow the sensitivity analysis to guide future actions aimed at reducing, minimizing, or even eliminating GHG emissions whenever possible.

The procedure was applied to the following parameters: (x) hemp productivity in the field, (y) the addition of binder to the pelleting process, and (z) the distance from the transportation of the PP resin production unit to the composite processing site. In addition to assessing the individual effect of each variable on the GWP, the sensitivity analysis also enabled an examination of the potential amplification or damping effects resulting from their associations.

For this purpose, a matrix system adapted from experimental planning practices was created, where the lowest contribution is coded as [−1], the intermediate stage as [0], and the most significant contribution as [+1]. This strategy yielded the indicator GWP (x, y, z), for which (x) and (z) can vary between [−1; +1], while the variable y can only range from [0; +1]. An explanatory summary of the foundations adopted for the sensitivity analysis is presented in Table 4.

## 3. Results and Discussion

### 3.1. GWP Associated with Subsystem 1: Hemp Stalks

**Figure 7:**
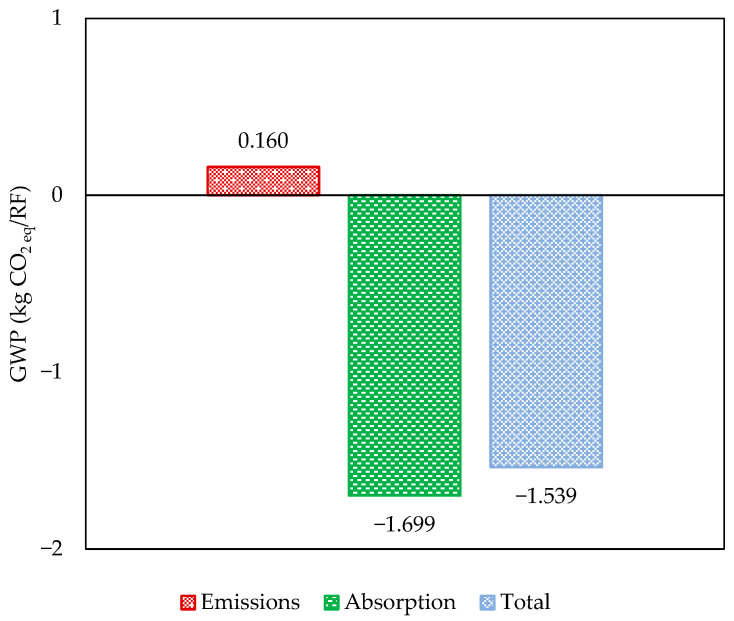
GWP related to hemp stalk.

Figure 7 presents the GWP values obtained for producing 1.0 kg of hemp stalks in bales. The cumulative value of −1.54 kg CO_2 eq_ indicates a positive carbon capture potential outcome. This performance results from carbon dioxide fixation by hemp plantations (CO_2_ uptake) through photosynthetic biological processes, which exceeds the total impacts of GHG emissions from Subsystem 1 by more than ten times; this means that the use of this product as an input in the supply chain of other products has the potential to reduce their CO_2 eq_ emissions by a proportion of 1.54 kg CO_2 eq_ for each kg of stalks applied in the process. In theory, this benefit could vary from eight to about thirteen times, depending on whether the values of the cultivars planted in Waterloo resemble those achieved by the hemp varieties studied in [27,33], which were 1.29 and 2.10 kg CO_2_/kg biomass, respectively (Table 1).

In this context, the adaptability of the Cannabis sativa species to local conditions is critical. Using animal manure for soil fertilization had a significant impact on the environmental performance of Subsystem 1. This approach notably reduced the environmental burdens associated with the synthesis of chemical fertilizers, enhancing overall performance by utilizing revalued waste from agricultural activities as a substitute. Therefore, at least in terms of the GWP, hemp biomass appears to be a promising intermediate for its intended uses.

**Figure 8:**
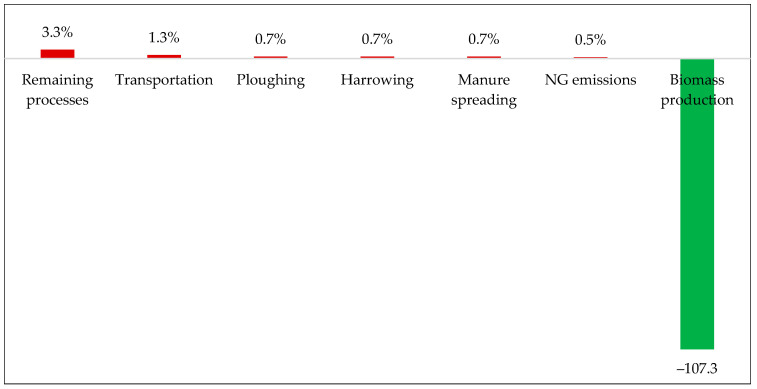
Subsystem 1: stage contributions to the GWP.

Figure 8 illustrates the contributions of the most significant impact factors to the GWP associated with hemp stalk production. As a result of the CO_2_ fixation rate, these values are relativized in relation to the overall performance of Subsystem 1. Upon examining the series in detail, it is observed that transportation constitutes the primary source of the impact (1.3%), owing to the distribution of agricultural materials (29 km) and seeds, which are administered at a rate of only 33.6 kg per hectare yet need to travel from Winnipeg (1996 km) by road to supply the crop. Following this, agricultural operations such as plowing, harrowing, and manure application contribute equally (~0.74%), as do the direct emissions of natural gas (NG) tied to the extraction and refining of petroleum, which will be converted into diesel oil. The other operations or processes comprising Subsystem 1 have individual shares of less than 0.5%, and, collectively, they account for approximately 3.3% of the impact on the GWP of the arrangement. Despite their specificities, the results are similar to those obtained by other authors [42,43], where the carbon captured by the plantation was found to be more than ten times greater than the CO_2 eq_ emissions, as both studies point out that the main factor responsible for the positive GWP values is the use of agricultural machinery.

### 3.2. GWP Associated with Subsystem 2: Hemp Pellets

**Figure 9:**
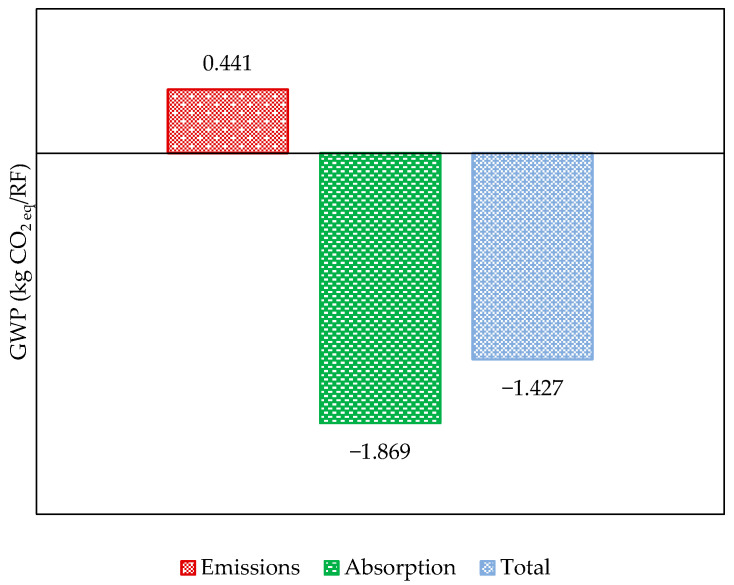
The GWP related to hemp pellets.

After estimating the GWP of hemp pellet manufacturing (Figure 9), we observe that, while still positive, this impact is less favorable than that estimated for Subsystem 1. A beneficial magnification effect is associated with CO_2_ capture due to processing losses, as the process consumes hemp fibers at a rate of 1.10 kg per kilogram of pellet. However, the share of GHG emissions in this stage of the Product System also rises significantly, resulting in an impact of 441 g CO_2 eq_/kg.

**Figure 10:**
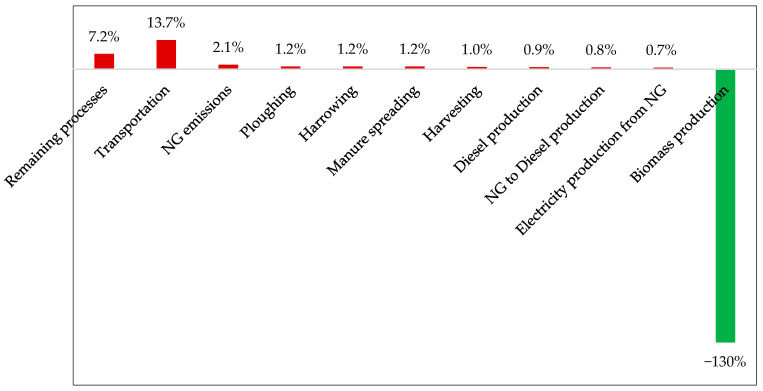
Subsystem 2: stage contributions to the GWP.

Figure 10 outlines the processes and operations that contribute most to the GWP associated with pellet production. In this case, transport is also responsible for significantly affecting the system’s performance, accounting for 14% of the contributions to the impact category. In this context, it is noteworthy that hemp bales are driven by road from the plantations to the pelletizing unit, covering a distance of approximately 1000 km.

Then there are the contributions arising from the extraction and processing of natural gas, which primarily manifest in the form of CH_4_ emissions into the atmosphere. They must be summed up in the loss of fossil CO_2_ into the atmosphere caused by burning the same fuel to supply thermal energy for pelletizing. Moreover, CH_4_ and CO_2_ emissions from oil extraction and refinement for diesel oil production must also be attributed to natural gas. Overall, the life cycle of natural gas, from the cradle to grave, contributes 42.6 g CO_2 eq_ of impact to the GWP for each kilogram of pellet produced. Lastly, agricultural operations (plowing, harrowing, manure application, and harvesting) continue influencing the pellets while hemp fibers are processed. This is precisely because the LCA technique employs a systemic assessment approach. In Subsystem 2, these transformations contribute 65.8 g CO_2 eq_/kg to the GWP. Additionally, it was decided to recalculate the environmental impact of hemp pellet production by considering a reduced distance between hemp bale production and pellet processing.

In this case, the calculations were performed for a distance of 100 km, as transportation is the stage in the production chain that contributes the most to the environmental indicator. The GWP was −1.604 kg CO_2 eq_/kg, representing a reduction of 12.4%. Therefore, it is evident that reducing the transportation needs for Subsystem 2 can significantly decrease the product’s GWP and should thus be encouraged.

It is worth noting that the process was optimized to produce short hemp fibers using the newly developed microdecortication method. Its strength lies in improved efficiency and smooth integration with the supply chain. Specifically, microdecortication is much more efficient for producing short hemp fibers, with yields of 85% or higher, compared to traditional decortication, which yields about 20% long fibers and requires additional energy to cut them into short fibers. Additionally, the densification (pelletization) process was optimized to lower the bulk density while maintaining the pellet strength for transportation and ensuring good dispersion during compounding. This marks significant progress in integrating with the supply chain because pellets are easier to transport, store, and feed into extruders than loose fiber. Further supply chain integration was achieved by optimizing the composite formulations to meet technical specifications similar to those of existing products (polypropylene glass fiber composite). This allows for the introduction of renewable feedstock without altering the final product design while also reducing the carbon footprint.

### 3.3. Comparison of Environmental Performance Between Conventional and Alternative Composites

A comparison of the environmental performance of alternative and conventional composites leads to two conclusions. While hemp pellets exhibit positive impacts regarding the GWP [−1.54 kg CO_2 eq_/kg pellet], transforming this material into a composite incurs sufficient environmental burdens to reverse this outcome, turning it into a detrimental production option for the environment in the same impact category (1.47 kg CO_2 eq_/kg composite).

**Figure 11:**
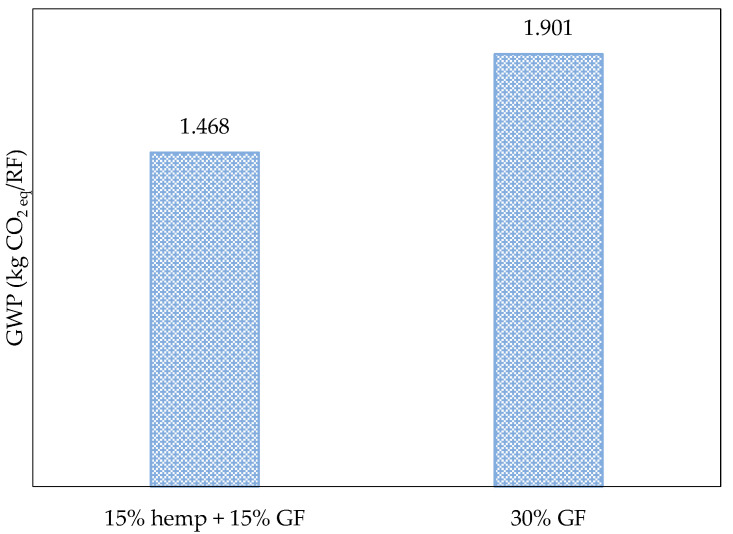
GWP values for alternative composites, including hybrid composites (short hemp and glass fibers) and traditional composites with glass fiber only.

Conversely, as shown in Figure 11, substituting 15% (w/w) of glass fiber with an equivalent amount of bio-based material in the composite formulation results in a 19% reduction in its GWP value. This relative reduction is almost the same amount obtained by La Rosa et al. [23], related to the partial replacement of GF with hemp fibers in a composite piece of piping, although the proportion of fiber and the polymeric matrix were not the same as those considered in this work. Le Duigou and Baley [44] made a comparison between PP composites with GF and natural fibers (in this case, flax) using the same PP/fiber ratio as the present study, and, regardless of the specificities of each approach, the GWP of the composite with natural fiber was reduced by approximately 10% compared to that of the material with GF. This reinforces the great potential of using natural fibers, such as hemp fibers, to reduce environmental burdens.

**Figure 12:**
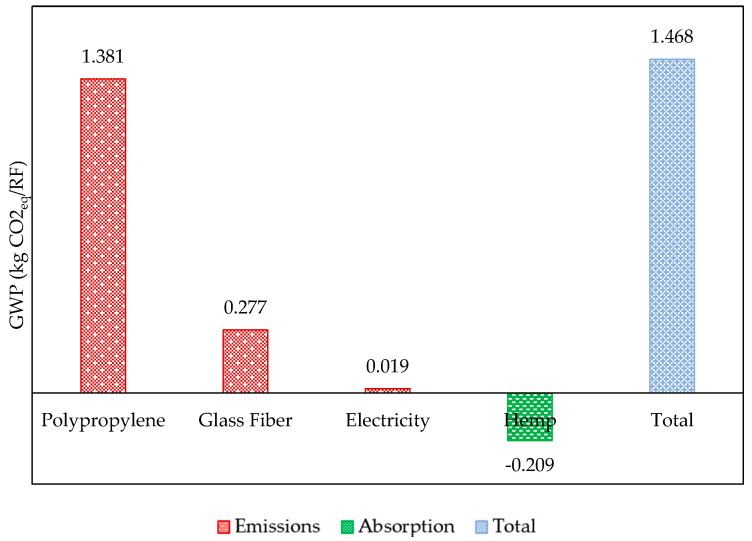
Absolute and individualized GWP values for each component of Subsystem 3 in the hybrid composite.

Hemp composite emissions can be more effectively analyzed when categorized into four components based on the distribution of environmental loads. These align with the life cycles, encompassing the range from “cradle-to-use” of the three active ingredients that make up the composite formulation (i.e., polypropylene, glass fiber, and hemp) and the setup designed for generating and transporting the electrical energy consumed during extrusion, where they are effectively transformed into the composite. Figure 12 presents the absolute and individualized GWP values of each component. According to these data, the life cycle of polypropylene contributes 94% of the impacts to the category, thus becoming the primary focus of attention for measures to mitigate this effect.

**Figure 13:**
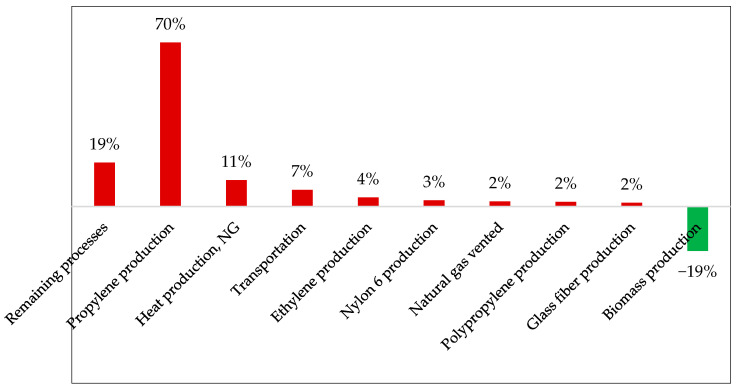
The contribution of each step in the hemp composite production process to the GWP.

We decided to explore this issue more thoroughly and discretize the contributions of the life cycle of composite production containing hemp fibers by its main processes. This partition is illustrated in Figure 13 as relativized values.

The analysis revealed that the processing of propylene (C_3_H_6_), a crucial input for obtaining PP, was the most significant contributor to GWP generation in the composite life cycle. The usual route for producing C_3_H_6_ is propane steam cracking, which occurs at temperatures around 850 °C [45,46]. Its contribution to the GWP of 1.02 kg CO_2 eq_/kg composite results from the CO_2_ emissions of fossil fuel combustion, particularly natural gas (NG), to produce the thermal energy needed.

The heat required to pelletize hemp fibers and the long-distance transportation also contribute to the impacts of the partially renewable composite, with 165 and 104 g CO_2 eq_/kg composite, respectively. Finally, it is crucial to emphasize the ability of hemp fibers to mitigate the environmental impacts produced during composite manufacturing without compromising their fundamental properties.

This characteristic further underscores the importance of intensifying efforts to enhance the role of this bio-based product in the value chains of these materials.

### 3.4. Findings of Sensitivity Analysis

The GWP results were categorized into two groups based on binder dosage to enhance the efficiency and clarity of the analysis. The first group did not consider the active ingredient dosage in its sensitivity analysis, resulting in GWP (x, 0, z) type outcomes. In contrast, the second group included binder addition, leading to GWP (x, +1, z) type estimates. These value sets are presented in Table 5 and Table 6 as absolute impact data.

Upon a closer evaluation of the GWP values, we found that the most significant impact difference for both series was 8.5%. Predictably, these variations occurred between scenarios of high agricultural productivity with shorter distances from polypropylene to composite processing plants, namely, GWP (+1, 0, −1) and GWP (+1, +1, −1) and the opposite scenarios of low-yield hemp cultivation with longer distances, namely, GWP (−1, 0, +1) and GWP (−1, +1, +1). While these fluctuations were minor compared to more important contributions to the GWP of the system, such as the impact of PP use, it is crucial to note that PP processing is not under the purview of composite manufacturers.

**Figure 14:**
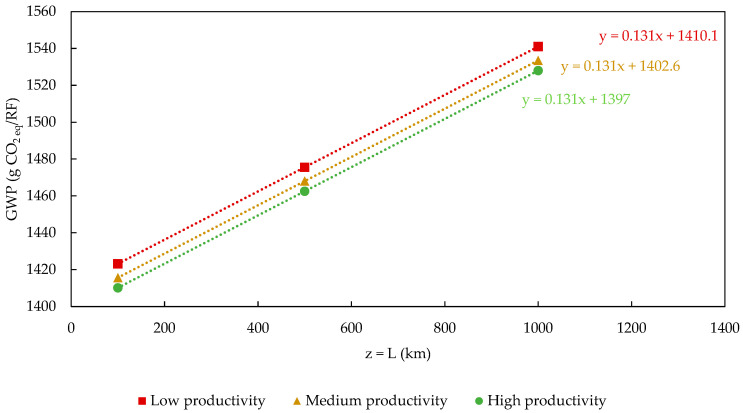
Sensitivity analysis of the GWP=f(L) variation when binder is not added:GWP(x,0,z).

**Figure 15:**
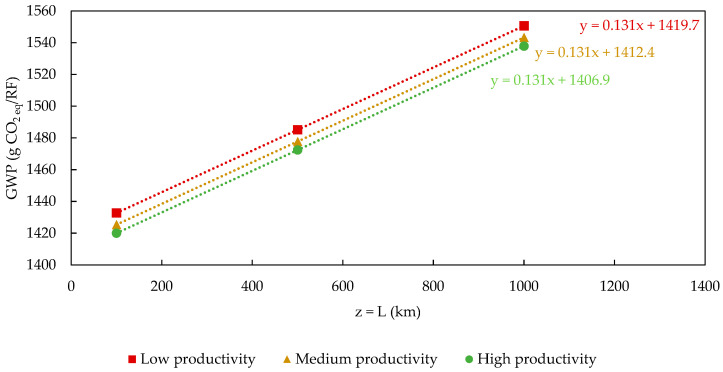
Sensitivity analysis of the GWP=g(L) variation when binder is added:GWP(x,+1,z).

Therefore, the only alternative left in this context would be to create a composite using a product similar to PP, which could affect the product’s properties and application range. Consequently, exploring alternatives that reduce environmental burdens in the operational areas of the Product System, before focusing on those indirectly linked to it, represents a highly effective strategy, even if it yields limited gains. An alternative to PP application is the use of bio-composites like polylactide (PLA) or poly-hydroxyl-butyrate (PHB) [42,43,47], as suggested by other authors [42,43,47]. Figure 14 and Figure 15 present approaches different to those previously adopted for the sensitivity analysis. In these instances, the results were also categorized into groups based on the addition of the binder. Once this categorization was made, curves representing the GWP as a function of L (GWP=f(L)) for each tested productivity level (P) during the analysis were constructed. The red curves and square markers (■) correspond to a scenario where P = 2471 kg/ha. The curves in yellow (▲) and green (●) correspond to productivity levels of 3707 and 4942 kg/ha. The results in Figure 14 and Figure 15 indicate that the variation in the GWP with the distance traveled between the PP and composite production plants is linear.

Furthermore, the contribution to the GWP from transportation, represented by the angular coefficients of all first-degree equations, is consistent across both groups of results, ranging from 13.1 g CO_2 eq_/RF for 100 km journeys to 131 g CO_2 eq_/RF for journeys reaching 1000 km. This characteristic, being common to both groups of values, should be expected, given the independent nature of the transportation distances and binder dosage.

Agricultural productivity related to hemp cultivation corresponds to one component of the linear coefficient in the same expressions. When comparing the results of these parameters within the same group of findings, it is evident, in a predictable manner, that agricultural productivity has an inverse influence on the overall GWP result of the composite. For the cases of GWP (−1, 0, z) and GWP (−1, +1, z), where P = 2471 kg hemp/ha, the linear coefficients reach their maximum values, while in the opposite scenarios, GWP (+1, 0, z) and GWP (+1, +1, z), where P = 4942 kg/ha, they reach their minimum values. Another significant aspect of agricultural productivity is that its maximum contribution to mitigating the Global Warming Potential is 13.0 g CO_2 eq_/RF for either set. This value is derived from the difference between the linear coefficients of the equations for maximum (●) and minimum (■) productivity. Therefore, considering the similarity between values, achieving a maximum level of productivity in hemp cultivation would imply the ability to transport polypropylene an additional distance of up to 100 km compared to minimum productivity without affecting the accumulated GWP result of the analyzed arrangement.

Finally, when employing this approach for a sensitivity analysis, we observe that the contribution of binder addition to the GWP of the composite’s life cycle is relatively modest (~9.5 g CO_2 eq_/RF). This value can be determined by the difference between the linear coefficients of the equations for the relationship GWP = f(L) with and without the addition of the active ingredient at the same level of agricultural productivity, that is, GWP (x_i_, 0, z_i_) and GWP (x_i_, +1, z_i_), respectively.

## 4. Recommendations

The analysis of the results of the products of interest gave rise to recommendations that, when applied, could improve their environmental performance. It is essential to remember that these suggestions aim exclusively at reducing contributions to the Global Warming Potential.

Given that manure transportation is one of the operations that contribute the most to the GWP of Subsystem 1, it is recommended to seek suppliers of this input located closest to the place of application. Considering that the manure used as a primary macronutrient during fertilization may have different origins (e.g., bovine, swine, or equine), proximity to the hemp cultivation area should be used as a criterion for selecting supply sources, provided that the characteristics and properties of the material meet the soil’s nutritional needs.

Another action that can mitigate these effects is using electricity from renewable sources, such as hydro, photovoltaic, or wind power, to drive machines and equipment. In addition, if discontinuing the use of binders does not result in losses for the composite synthesis process or alter its properties to the point of affecting applications, a natural path would be to use only water in pelletization.

Additionally, for this stage of production arrangement, it is recommended to intensify the use of hemp biomass in the formulation of the composite, incorporating it as a replacement for PP. However, this guideline must be followed carefully and cautiously to avoid changing the product specifications, making its use unfeasible or restrictive. Transportation significantly affects the GWP of the composites. Therefore, it is recommended that the logistics for distributing inputs and raw materials in these transformations be optimized, with a focus on environmental performance.

While this research focused on replacing glass fiber with hemp biomass, polypropylene (PP) had a significant influence on the Global Warming Potential (GWP) of the final material. Thus, a review of the polymer matrix to decrease its role in the structure, possibly by adding another active ingredient, could be a viable approach. Materials that produce lower greenhouse gas (GHG) emissions, such as recycled or renewable polymers, could be investigated. However, when applying a Life Cycle Assessment, it is possible to assess the environmental impacts of these materials, including resin production and the cultivation of biomaterials.

Continuing with the same approach, although the GWP is a commonly used metric to describe the environmental profile of many human-made systems, future research should be expanded to include other environmental impact categories, such as acidification potential, eutrophication potential, and ozone depletion potential, which are typical of life cycles related to agricultural and industrial activities. Implementing this strategy will offer a more comprehensive understanding of the environmental sustainability of hemp fiber composites, supporting more informed decision-making in this field.

Finally, another aspect to consider is that the research presented in this manuscript uses data collected from hemp farming under normal conditions across several acres and from manufacturing equipment. The equipment used for data collection is suitable for small-scale commercial operations; however, it can be scaled up for larger operations. As a result, the technology can be expanded to increase production as commercial operations grow while maintaining or even improving energy efficiency, thus further reducing the environmental impact. However, it is essential to note that, while efficiency improvements are likely to occur with an increased manufacturing scale, the core technology may remain unchanged; therefore, the assumptions used to evaluate the environmental impact do not require significant adjustments.

## 5. Conclusions

After applying the Life Cycle Assessment technique to composites containing hemp and its intermediates—hemp stalks in bales and hemp pellets in bags—it was concluded that the partial replacement of synthetic fibers with biomass fibers in a composite offers significant environmental advantages. This reduced the product’s Global Warming Potential (GWP) by up to 25% without compromising its mechanical properties.

Additionally, the most notable individual contribution to the GWP indicator was associated with polypropylene, another raw material used in the manufacturing process. In Subsystem 1, the amount of CO_2 eq_ absorbed during the plantation growth phase far exceeded the emissions related to soil preparation and the processing of hemp stalks, indicating that an increase in productivity from 2471 kg/ha to 4942 kg/ha does not substantially alter the GWP value per kg of product produced. Another finding revealed that using a binder in the pelletizing process raised the GWP of the composite by only 0.63%.

From a logistics standpoint, transportation significantly contributed to the higher GWP of hemp stalks and hemp pellets, so minimizing the distances traveled by the inputs of these products could help decrease GHG emissions.

Finally, this study demonstrated that both the production of hemp stalks in bales and the production of hemp pellets using the newly invented microdecortication process exhibited a negative GWP, irrespective of the productivity of hemp in the field or the addition of binder during the pelletizing process. Therefore, their application as inputs in other value chains holds excellent potential for reducing product emissions.

## Figures and Tables

**Table 1:** Literature values for CO_2_ uptake rates by biomass during hemp cultivation.

Reference	Reference CO_2_ Uptake Rate (kg CO_2_ /kg Biomass)
[27]	1.29
[28]	1.47
[29]	1.53
[30]	1.73
[31]	1.84
[32]	1.85
[33]	2.10
**Average value**	**1.69**

**Table 2:** Variation ranges in electrical energy consumption for operations involved in the production of pellets and hemp.

Operation	Electrical Power (kW)
Cutting	0.80–4.00
Classification	0.75–5.00
Milling	15.0–50.0
Fine collection	10.0–18.0
Pelletization	9.00–16.0
Cooling	0.50–3.50
Belt conveying	5.00–12.0

**Table 3:** Mechanical characterization of composite materials.

Sample	Relative Composition (% *)	Tensile Modulus (MPa—23 °C)	Flexural Modulus (MPa—23 °C)	Impact Notched (kJ/m^2^—23 °C)	Density (g/cm^3^)
30GF–PP (regular)	30% GF + 70% PP	4000 ± 514	4000 ± 232	6.00 ± 0.15	1.21 ± 0.23
15H–15GF–PP (alternative)	15% hemp + 15% + GF + 70% PP	4648 ± 159	4364 ± 208	5.10 ± 0.59	1.05 ± 0.42

Legend: H: hemp pellet; GF: glass fiber; PP: polypropylene; * mass basis (data provided by Tangho Green Canada and University of Waterloo).

**Table 4:** Synoptic table of the parameters and their coding involved in the sensitivity analysis.

Parameter	Hemp Productivity	Addition of Binder	Distance Between Plants (PP—Composite)
	(x)	(y)	(z)
Code/Unit	(kg/ha)	–	(km)
−1	2471	–	100
0	3707	No	500
1	4942	Yes	1000

**Table 5:** Global Warming Potentials for the situation type $GWP\left(x;0;z\right)$.

Addition of Binder:(y) = 0				
Parameters/Codes		Hemp Productivity: (x)		
		−1	0	+1
Spacing between plants: (z)	−1	1.423	1.416	1.410
	0	1.476	1.468	1.463
	+1	1.541	1.534	1.528

**Table 6:** Global Warming Potentials for the situation type GWP(x;+1;z).

Addition of Binder:(y) = +1				
Parameters/Codes		Hemp Productivity: (x)		
		−1	0	+1
Spacing between plants: (z)	−1	1.433	1.425	1.420
	0	1.485	1.478	1.472
	+1	1.551	1.543	1.538

## Data Availability

The original contributions presented in this study are included in the article. Further inquiries can be directed to the corresponding authors.

## Abbreviations

The following abbreviations are used in this manuscript:

GWP	Global Warming Potential
GF	Glass fiber
PP	Polypropylene
LCA	Life Cycle Assessment
DfE	Design for Environment
MAP	Monoammonium phosphate
MCC	Mass contribution criterion
CMC	Cumulative mass contribution
CEC	Cumulative energy contribution
ON	Ontario, Canada
RF	Reference flow
OLR	Open-loop recycling
IPCC	Intergovernmental Panel on Climate Change
NG	Natural gas
